# The Role of Unobtrusive Home-Based Continuous Sensing in the Management of Postacute Sequelae of SARS CoV-2

**DOI:** 10.2196/32713

**Published:** 2022-01-26

**Authors:** Benjamin Harris Peterson Corman, Sritha Rajupet, Fan Ye, Elinor Randi Schoenfeld

**Affiliations:** 1 Renaissance School of Medicine Stony Brook University Stony Brook, NY United States; 2 Program in Public Health Stony Brook University Stony Brook, NY United States; 3 Department of Family, Population & Preventive Medicine Renaissance School of Medicine Stony Brook University Stony Brook, NY United States; 4 Department of Biomedical Informatics Renaissance School of Medicine Stony Brook University Stony Brook, NY United States; 5 Department of Electrical and Computer Engineering College of Engineering and Applied Science Stony Brook University Stony Brook, NY United States

**Keywords:** SARS CoV-2, COVID-19, post-acute sequelae of SARS CoV-2 (PASC), post-COVID, long COVID, continuous sensing, passive monitoring, wearable sensors, contactless sensors, vital sign monitoring

## Abstract

Amid the COVID-19 pandemic, it has been reported that greater than 35% of patients with confirmed or suspected COVID-19 develop postacute sequelae of SARS CoV-2 (PASC). PASC is still a disease for which preliminary medical data are being collected—mostly measurements collected during hospital or clinical visits—and pathophysiological understanding is yet in its infancy. The disease is notable for its prevalence and its variable symptom presentation, and as such, management plans could be more holistically made if health care providers had access to unobtrusive home-based wearable and contactless continuous physiologic and physical sensor data. Such between-hospital or between-clinic data can quantitatively elucidate a majority of the temporal evolution of PASC symptoms. Although not universally of comparable accuracy to gold standard medical devices, home-deployed sensors offer great insights into the development and progression of PASC. Suitable sensors include those providing vital signs and activity measurements that correlate directly or by proxy to documented PASC symptoms. Such continuous, home-based data can give care providers contextualized information from which symptom exacerbation or relieving factors may be classified. Such data can also improve the collective academic understanding of PASC by providing temporally and activity-associated symptom cataloging. In this viewpoint, we make a case for the utilization of home-based continuous sensing that can serve as a foundation from which medical professionals and engineers may develop and pursue long-term mitigation strategies for PASC.

## Introduction

### Postacute Sequelae of SARS CoV-2

On March 11, 2020, the World Health Organization (WHO) declared COVID-19 a pandemic. Since then, the responsible virus, SARS-CoV-2, has spread across the international community, leading to the most pervasive and defining public health event of this generation. In the United States, as of this writing, there have been over 65 million cases and nearly 850,000 deaths [1]. Less than 1 year after COVID-19 was declared a pandemic, the Food and Drug Administration (FDA) issued Emergency Use Authorization for the Pfizer-BioNTech, Moderna, and Janssen COVID-19 vaccines [2]. Amid the ongoing US nationwide vaccination campaign, attention has turned to the allocation of resources to address the long-term health impacts of the pandemic.

The medical community has paid particular attention to those individuals whose COVID-19 symptoms never fully remitted, have since recurred, or have developed new symptoms thought to be related to having COVID-19. Current estimates are that upwards of 35% of COVID-19 patients fall into this group of individuals who have a disease now recognized formally as postacute sequelae of SARS CoV-2 (PASC) but is also colloquially being referred to as post-COVID, long COVID, long-haul COVID, postacute COVID-19, long-term effects of COVID, or chronic COVID [3,4]. This can translate to over 15 million PASC patients utilizing the health care system in the United States alone, with the numbers growing daily.

PASC occurs in any patient with a prior COVID-19 infection, yet the severity of the disease proves to be a poor predictor of prolonged or recurrent symptoms [3]. PASC patients present with a variety of multisystem symptoms that have made evaluation, diagnosis, and treatment more challenging. We present here a proposal for the widespread adoption of continuous sensing devices to provide contextualized surveillance data for patients’ core vital signs and activities between medical encounters (eg, at home). Such continuous sensing and the subsequent raw data processing would provide quantitative means and insights into the symptom progression of PASC at home and between hospital or clinical visits. They will enable clinicians to holistically assess standards of care while simultaneously offering higher levels of operationalism to the management of PASC. We also provide suggestions on how to interpret such data in the context of PASC management. Evaluations of these data at the population level can serve as the foundation for the creation of predictive analytics tools that can be utilized in the management of all PASC patients. It must be stressed that, while we see substantial benefit in the use of continuous monitoring for PASC and chronic diseases in general, we do not intend to diminish the importance of current efforts to mitigate the acute effects of the pandemic, and such home-based sensing serves to complement and not replace the rigorous medical device–based data collection performed during hospital or clinical visits. Instead, our goal in this paper is to propose a framework from which medical professionals and engineers may pursue long-term mitigation strategies for PASC via home-based continuous sensing data in conjunction with ongoing medical and technical innovations and efforts in the acute care setting for COVID-19.

### Background

A particular challenge with managing PASC is the fact that there has yet to be an established standard of care. Until recently, research on the topic could be described as sparse and grassroots, with patient reports of persistent symptoms, particularly those from disparate PASC support groups, driving clinical understanding [5]. PASC is now widely recognized by the medical community, with the National Institutes of Health (NIH) launching a broad-based initiative to understand the disease and the long-term health of this cohort of patients [6]. In a recent retrospective cohort study, it was found that 36.55% of all COVID-19 patients have at least one PASC-associated symptom between 3 and 6 months after their initial COVID-19 diagnosis [4]. Such an assessment captures the breadth of this public health issue and represents a substantial sustained burden on patients, families, communities, medical care systems, and the nation.

Despite the formal recognition of PASC, a notable lack of understanding of the pathophysiology of the syndrome remains. Although data from spirometry, electrocardiography, auscultation, stress tests, and other clinical measures can assist with developing a clinical picture of COVID-19’s impact on the patient’s lungs and heart, these tests represent point observations. Symptoms of PASC, however, have been found to wax and wane over time, necessitating continuous monitoring for accurate quantitative measures of symptoms [7]. Furthermore, it has been observed that some symptoms are exacerbated during the performance of specific daily tasks [3,8]. As these activities most often occur outside of the clinical setting, it is vitally important that data should be collected in a home (or home-like) environment to identify these potentially transient events.

Currently, in research and clinical studies, a popular method by which these data can be collected is by means of a symptom diary in which patients self-report changes in their physiological state. Such tools rely on retrospective reporting, making them prone to recall bias in addition to self-serving biases [9]. Diaries also pose a significant respondent burden, and as such, adherence to daily diary keeping is a significant consideration. In one study that required chronic pain participants to record 3 pain entries per day for 21 consecutive days, it was found that only 10.9% of traditional paper diary entries were recorded within the temporally defined compliance window set by the study coordinators, despite reported compliance on 90.5% of entries [10]. It has been found that there is limited evidence to suggest significant clinical utility in traditional symptom diaries, with one study concluding that among asthmatic children there was no statistically significant beneficial effect of keeping a symptom diary on day-to-day asthma control nor was there a decrease in the future risk of asthma control [11]. Although electronic diaries with measures designed to enhance use compliance have been shown to be associated with greater adherence to the temporal demands of keeping a symptom diary in some (but not all) contexts, there remains a significant user burden. As such, there remains nonuniversal adherence, and the utility of the collected data in informing clinical decisions with beneficial outcomes is an area of ongoing research [9,10,12,13]. As such, there is a pressing need for a method of tracking symptoms that is standardized between patients, quantitative in its collection, and poses minimal patient or user burden. Sensing technologies, particularly those that are unobtrusive in nature, offer a promising solution to all 3 of these requirements.

Although there is a precedent for providing patients wearable sensors for vital sensing between hospital encounters, most notably in the form of Holter monitors for arrhythmia detection, their adoption has been far from widespread in the context of PASC, and the conspicuousness and complexity of these sensors limit their usability in all scenarios for extended periods [14,15]. A more user-friendly solution to long-term monitoring involves unobtrusive technologies that lend themselves to continuous passive use across a range of activities and whose output data is sufficiently robust to provide a reasonably accurate physiological picture.

Continuously monitoring sensors are already being implemented in the context of a multitude of chronic diseases including chronic obstructive pulmonary disease, asthma, congestive heart failure, and many more [16-19]. The use of such sensors in the context of PASC is an important tool for disease management. However, it has the additional benefit of providing substantial data for researchers to develop predictive analytics tools for PASC, making it an incredibly powerful tool for elucidating the relatively data-poor academic space of COVID-19 sequelae.

In the consumer market, there currently exist 2 primary means by which continuous vital sign data can be obtained: by way of wearable sensors and by way of contactless sensors. Wearable sensors require physical proximity (usually direct touch to the human body) between the user and the sensor [20]. This is the more pervasive sensing technology currently in the consumer market, with smartwatches, chest-strap heart rate monitors, glucometers, and smart clothing comprising some of the products in this family of technologies. Wearable sensors tend to be more portable and generally more accurate than contactless sensors but require users to charge and wear them regularly for accurate and consistent data collection.

Contactless sensors, by contrast, do not require physical proximity to a user [20]. Instead, they can collect data from a distance, without touching the human body. These sensors can function completely passively with no additional user efforts (ie, charging, wearing) beyond the initial setup. These sensors can be mounted to a wall or placed on a surface. However, while they have the benefit of operating with minimal user efforts, they lack portability. We believe it is with widespread utilization of a combination of wearable and contactless sensors that PASC pathophysiology can be best understood and management strategies can be best implemented. Although both sensor families offer complementary insights in disease tracking and managing and can further inform a clinician’s understanding of the disease, it must be stressed that these sensors do not represent a replacement for existing gold standard instruments. Instead, sensor findings may prompt a clinician to perform further workup.

With regards to PASC diagnosis, there currently exists no single diagnostic criterion for PASC. The National Institute for Health and Care Excellence (NICE) in the United Kingdom defines “post-COVID-19 syndrome” as signs and symptoms that develop during or after an infection consistent with COVID-19, continue for more than 12 weeks, and are not explained by an alternative diagnosis [21]. In the United States, such a universal definition that can serve as the basis for diagnoses, even one as open-ended as that put forth by NICE, does not exist. Although not intended for the basis of a diagnostic criterion, the WHO recently put forth their own definition of the disease that was comparably vague in its description, describing a “condition [that] occurs in individuals with a history of probable or confirmed SARS-CoV-2 infection, usually 3 months from the onset of COVID-19 with symptoms that last for at least 2 months and cannot be explained by an alternative diagnosis” [22]. Similarly, the US Centers for Disease Control and Prevention (CDC) recognizes PASC as “a wide range of new, returning, or ongoing health problems people can experience four or more weeks after first being infected with the virus that causes COVID-19” [23]. As such, it is up to the clinician’s discretion as to how and when to give a patient a diagnosis of PASC. Although continuous sensing will not change the lack of unified diagnostic criteria, it will provide a framework to quantitatively track signs and symptoms associated with PASC from the initial COVID-19 diagnosis (or suspected diagnosis) to the treatment of the acute phase and forward.

When treatment is indicated in the management of PASC, it is in the context of supportive management and symptom monitoring [24]. It is, therefore, important that medical providers are given data to inform the best medical treatment and to track patient progress. A symptom-specific means of continually monitoring a patient can provide quantitative measures to suggest symptom abatement or the efficacy of treatment.

## Quantitatively Measurable PASC Symptoms

### Symptom Presentation

The presentation of PASC is variable. Symptoms may be constant, or they may wax and wane. Generally, care providers obtain physiological measures of their patients at the time of clinical visits. Given that disease presentation is inconstant between patients and across time, having physiological measurements obtained with sensors between clinical visits would provide health care professionals a more holistic clinical picture.

Large-scale studies specifically designed to identify and catalog symptoms have been and are being conducted based on patient-reported symptoms [4,6,25-31]. Although each has some variability in the measured relative prevalence of symptoms, fatigue and shortness of breath are generally thought to be the most common symptoms [32]. Although further prevalence data are discrepant between studies, most common complaints of PASC involve the following systems or combinations thereof: respiratory, neurologic, psychiatric, metabolic, cardiovascular, gastrointestinal, musculoskeletal, and general well-being, as well as some less commonly identified other sequelae [3,25]. We provide in the following paragraphs a summary of pathophysiologic burdens associated with PASC (either via direct causal association stemming from COVID-19 or due to preexisting conditions acting as a risk factor for the development of disease). We also offer potential sensor modalities whose implementation between medical encounters may directly or by proxy provide quantitative data to evaluate physiological changes associated with each respective symptom. A summary of PASC-associated symptoms and the potential sensing technologies that may quantify them is provided in Table 1 [29].

**Table 1:** Suggested sensor modalities to be used for the quantification of a selection of common postacute sequelae of SARS CoV-2 (PASC) symptoms.

Condition	Sensor modality
Acute coronary disease	Pose/motion monitor, activity monitor, pulse oximeter, heart rate
Anxiety	Sleep perturbations, activity monitor
Arrhythmia	Heart rate/rhythm monitor
Brain fog	Activity monitor
Changes in menstrual cycle	Heart rate monitor, temperature monitor
Chest pain	Activity monitor, pose/motion monitor
Constipation	Pose/motion monitor, activity monitor
Coughing	Microphone, pose/motion monitor
Depression	Sleep perturbations, activity monitor
Diabetes	Blood glucose monitor, insulin monitor, blood pressure monitor
Diarrhea	Pose/motion monitor, activity monitor
Fatigue	Sleep perturbations, activity monitor
Fever	Temperature monitor
Forgetfulness	Activity monitor
Gastroesophageal reflux disease	Heart rate, respiratory rate, pose/motion monitor
Headaches	Activity monitor
Heart failure	Heart rate/rhythm monitor
Hypoxemia	Pulse oximeter
Joint pain	Pose/motion monitor, heart rate, respiratory rate, activity monitor
Lightheadedness	Activity monitor, pose/motion monitor
Lipid metabolism disorders	Activity monitor, blood pressure monitor
Muscle weakness	Pose/motion monitor, heart rate, respiratory rate, activity monitor
Obesity	Weight tracking, activity monitor, blood pressure monitor
Olfactory perturbation	Activity monitor
Positional orthostatic tachycardia syndrome	Heart rate/rhythm monitor, pose/motion monitor
Posttraumatic stress disorder–associated symptoms	Sleep perturbations, activity monitor
Rash	Activity monitor, lesion identification
Shortness of breath	Pulse oximeter, respiratory rate
Stroke	Activity monitor, pose/motion monitor

### Respiratory Manifestations

Given the propensity of COVID-19 to cause debilitating respiratory symptoms, the utility of continuous monitoring is particularly apparent in the context of measuring lung function. Shortness of breath is one of the most common presenting symptoms in both acute COVID-19 and PASC [25]. Existing personal sensor technologies are well-equipped to monitor basic respiratory signs including respiratory rate. A recent case study demonstrated a management plan involving continuously measuring respiration and pulse rates and daily step counts using a clothing-adhered sensor that utilized photoplethysmography, tri-axis accelerometers, and a dedicated sensor of respiratory effort. The sensor was used to monitor the at-home recovery of a COVID patient with several weeks of persistent respiratory symptoms [33]. A recent article presented the reliability of a novel skin-adhered strain sensor in measuring respiration rate and volume measurements [34]. The study demonstrated an ability to extrapolate volume and rate data from these sensors to coarsely predict spirometry results. Pulmonary function tests like spirometry are important in the context of PASC as both restrictive and obstructive patterns of lung function have been reported among PASC patients [35].

Beyond shortness of breath, hypoxemia is a common respiratory complaint at 6 months following a positive COVID-19 diagnosis [25]. Pulse oximetry monitoring has long been a focus of personal wearable sensors. Fitbit, Apple, Garmin, and other smartwatch manufacturers have recently begun incorporating pulse oximetry as standard product features. These products are a complement to existing finger-based pulse oximeters whose utility as continuous monitoring devices are limited by size and inconvenience to the conduct of daily activities. Exertional dyspnea, the shortness of breath that occurs in the context of physical exertion, can be quantified with sensors that can track pulse oximetry in conjunction with markers of physical activity including heart rate, respiratory rate, or activity monitoring. Such a quantifiable measure of exertional dyspnea would allow for the early identification of patients in need of pulmonary or cardiac evaluation and the early initiation of therapeutic interventions [36-38].

Cough is another common PASC complaint. Current technologies exist that utilize continuous microphone-collected ambient sound to identify coughs using classification algorithms [39]. This offers a reasonable cost and resource-effective means of tracking coughing events across a spectrum of chronic diseases including PASC.

### Cardiovascular Manifestations

In the context of PASC, cardiovascular symptoms and conditions of particular prevalence include chest pain, acute coronary disease, arrhythmias, and heart failure. Chest pain may be of cardiac, pulmonological, gastrointestinal, nervous, or musculoskeletal origin, but obtaining signs via proper continuous sensing can provide a means by which these etiologies can be identified and treatment can be most effectively directed and monitored.

Many of these heart conditions lend themselves particularly well to continuous sensing in ways that noncardiovascular chest pain may not [40-42]. Arrhythmias including sinus tachycardia, bradycardia, atrioventricular blocks, fibrillations, and flutters can all be picked up with a sufficiently sensitive heart rate monitor. These come standard in many commercial smartwatches, many of which now have primitive electrocardiogram capabilities that can theoretically detect symptoms of acute coronary disease before it manifests in myocardial infarction.

Additionally, some PASC patients report a condition called postural orthostatic tachycardia syndrome (POTS) [43]. Patients with POTS experience tachycardic symptoms in the context of postural change. As a result, a continuous, contextualized means of collecting postural and heart rate data would be integral to developing a full picture of the symptoms and their manifestations.

### Psychiatric Manifestations

Many with PASC report psychiatric symptoms including feelings of anxiety and depression, with some patients having comorbid diagnoses of posttraumatic stress disorder. As with many of these symptom manifestations, there is not a single way by which continuous monitoring may be able to quantify these. There are, however, a multitude of modalities through which an adept care provider may make clinical recommendations. One such means by which diagnoses may be informed and treatment can be monitored is in the context of sleep disturbances. Many commercially available freestanding and smartphone-based contactless sensors as well as wearable devices including popular smartwatches track sleep quality, with some devices even offering the ability to stage sleep [44]. Assessing changes in quality, timing, duration, or staging of sleep can be useful in identifying these psychiatric symptoms and providing data to guide clinical intervention. Additionally, one particularly promising means by which psychiatric manifestations may be tracked is via activity and behavior changes. Sensors that can detect fine granularity changes in body pose, motion at seconds scale, and daily activities at minutes scale over time may elucidate psychiatric symptoms (though other symptom profiles may manifest in activity and behavior changes as well and thus need to be differentiated with high fidelity) [18,45]. These include wearable kinematic sensors or contactless depth sensors that can measure the coarse-grained 3-dimensional contours of surrounding objects, including the positional movement of users [46,47].

### Neurologic Manifestations

Some of the symptoms commonly associated with PASC that are most prominently featured in the lay media are neurologic manifestations, particularly so-called “COVID-19 brain fog.” Many treatments have been purported to help “clear” brain fog, but its pathophysiology is, as of yet, poorly understood, and the home remedies suggested are far from universally effective [48]. Such a dearth of understanding necessitates the use of tools to actively monitor neurologic symptoms. Behavior monitoring, as proposed in the previous section, using kinematic sensors or depth-sensing devices may be effective at quantifying changes in neurologic status.

Neurologic manifestations of PASC that may manifest in behavior change in addition to brain fog include stroke, lightheadedness, memory problems, headache, and olfactory perturbations [25]. It has been reported that changes in blood pressure, body temperature, blood glucose, and blood oxygen saturation may also be associated with strokes. Thus, although not specific for neurologic involvement, tracking these vital signs may be useful in identifying the onset or exacerbation of neurologic dysfunction [49]. Lightheadedness is a symptom whose presenting signs may be particularly useful in the setting of contextualized data. For example, lightheadedness spells may be quantified based on altered movement (ie, swaying, or slow, purposive movement) in the context of rapid changes in body position as might occur with a sudden rise from a seated position. For other neurologic manifestations, namely forgetfulness, headaches, and olfactory perturbations, physiological changes may not be easily measured but might be inferred indirectly through sensing of certain motions (ie, hands massaging the face or skull in the setting of headaches). Though such inference of symptoms based on body motion and activity alone might be unreliable, the ability of such sensor data to be retroactively interpreted by both a physician and the patient can help elucidate such basic metrics as date of symptom onset and frequency of recurrence as well as help identify exacerbating or relieving factors.

### Metabolic Manifestations

Metabolism is an area of vital sensing that has historically been difficult to track. Although metabolic manifestations have been less commonly implicated than other systems in the context of PASC, patients have an excess burden of disorders of lipid metabolism, obesity, and diabetes mellitus. It is not yet clear to what extent these metabolic conditions in particular are associated with the disease pathology itself and to what extent underlying metabolic conditions predispose patients to PASC [50-52].

There currently exist products that, although not continuous sensing devices, claim to assess metabolic end products in exhaled air to give indications about the user’s metabolic state [53]. Although an interesting proposal that demonstrates reasonable validity in initial studies, the product is yet to be widely tested and lacks FDA recognition.

Other potential sensing modalities include continuous glucose monitoring as has been widely used for those with diabetes mellitus. There have also been strides made towards developing flexible, adherent patches that utilize ultrasound to continuously measure blood pressure that can serve as a proxy for overall metabolic functioning [54,55]. Smartwatch-based blood pressure sensors are also in development, though the validity of such technologies has not been fully evaluated [56]. Slower-order changes like new or worsening obesity should be regularly monitored, but their slow progression may better fit the purview of interval monitoring, rather than continuous sensing. Smart scales and regular BMI measurements are better suited to quantify these trends.

### Gastrointestinal Manifestations

Although there is a lack of published evidence about the effectiveness of apps and sensors in tracking and managing gastroenterological care, adopting technologies to enhance patient care in this field has been and remains an active area of academic focus [57]. Current sensors that may be able to identify gastrointestinal manifestations of PASC (including those of constipation and diarrhea) include activity or presence sensors that track a user’s time in the bathroom. Gastroesophageal reflux disease, a reported manifestation of PASC, might be assessed with heart rate monitors and body pose sensors that can identify symptoms in the context of postprandial phases or while the user is lying down.

### Musculoskeletal Manifestations

Muscle weakness and joint pain have been reported among PASC patients, and the signs associated with them may be well quantified using heart and respiratory rate monitors or body pose sensors, particularly in cases for which there is a decreased range of motion. Pain that affects a patient’s ability to perform activities of daily living may manifest as behavior changes and can be assessed with activity monitors. Electrodermal conductance has been shown to increase in the setting of pain, so monitoring skin conductance could also provide insight into the pathophysiology of PASC [58,59]. Some work has also been done to correlate facial expressions with quantitative measures of pain [60].

### General Well-being Manifestations

Fatigue is an extremely common manifestation of PASC and is one of the most common presenting symptoms. It has been suggested that electroencephalogram (EEG) measurements can also be used to detect symptoms of fatigue, but until EEG technologies can be used discreetly by users, their use will likely be limited to controlled clinical and research settings [61]. In lieu of using EEG measurements, activity type and level can easily be measured to quantify fatigue symptoms, and while not specific for fatigue, they can be used by knowledgeable care providers in combination with other clinical measures to better understand a patient’s presenting signs. Such activity measurements can be made using either wearable or contactless solutions as presented in the previous sections [62,63].

### Other Reported Symptoms

Other reported symptoms of PASC that are recognized by the CDC that have not yet been presented in this viewpoint include fever, rash, and changes in menstrual cycles. Signs of fever can most directly be quantified using sensors such as infrared thermographic cameras [64]. New-onset rashes may be best temporally tagged with activity monitoring that identifies new or worsening instances of itching. Wearable sensor patches have been developed that can sense the act of itching and can be utilized in the setting of PASC [65]. Alternatively, cameras trained to recognize users may be able to identify new visible lesions, though such technology is currently far from being commercially or medically viable and alternative non-camera sensing is preferred as users have demonstrated concerns of poor privacy and discomfort with camera-based sensors [66]. Changes in menstrual cycles may be tracked, in part, using heart rate and/or temperature sensors during periods of sleep [67,68].

## Sensor Technologies

### Overview

**Figure 1:**
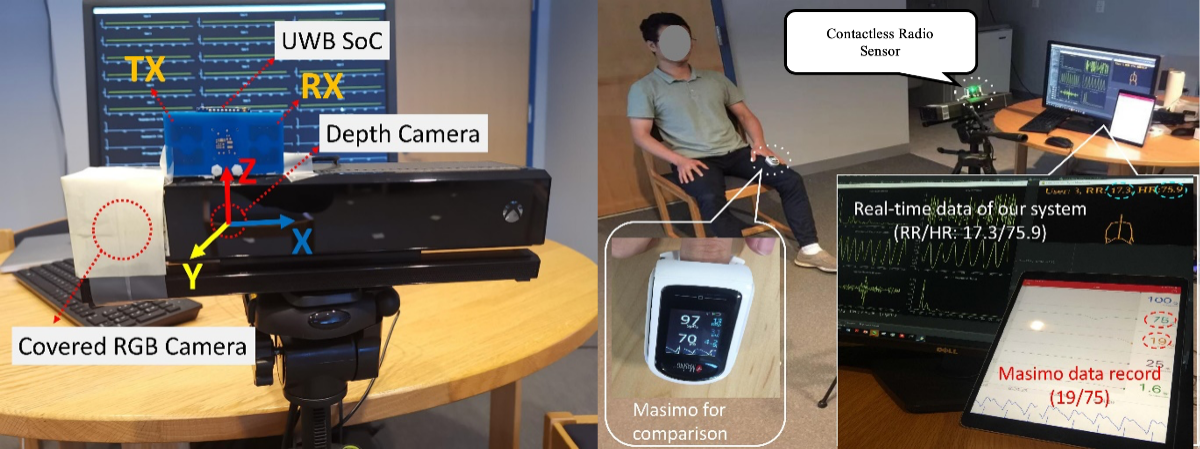
Illustration of a contactless sensor using an ultra-wideband (UWB) radio system on chip (SoC) and a depth camera. The radio waves reflect off the chest and become modulated by respiration and heartbeat movement and are received and processed in order to extract heart rate (HR) and respiration rate (RR). Such contactless sensors can produce results similar to fingertip oximeter pulse readings. This sensor is a prototype that we envision will be housed in a small-form customized hardware package. RGB: red, green, blue; TX: transmitting antenna; RX: receiving antenna.

Several continuous sensing technologies are currently available for consumer use, and more are in research or development. Currently, the majority of sensors fall under the category of wearables. These products, like the Apple Watch, the Fitbit Sense, or the Samsung Galaxy Watch3, have the benefit of being portable, though users must remember to constantly charge and wear the products to collect data. These pose physical and cognitive burdens to patients, and achieving long-term compliance can be difficult, especially for those with impaired dexterity or cognition. Aside from wearable sensors, there is growing interest in engineering circles to develop contactless sensors. Such sensors usually obtain measures without touching the human body and do not require any physical or cognitive efforts from the user. Figure 1 illustrates a prototype designed at Stony Brook University using off-the-shelf components, in particular an ultra-wideband radio that transmits extremely short pulses that reflect off the human body [69]. The chest movements from respirations and heart beats cause changes in the reflected signal. After sophisticated signal processing, the heart and respiration rates can be extracted (shown on the computer screen). Such a device can derive pulse data that are comparable to those of an FDA-approved fingertip oximeter (Masimo MightySat), whose data are shown on an iPad. The depth camera, a special device that generates coarse-grained 3-dimensional body joint locations but not detailed regular RGB images, can be used to recognize the body pose and thus activities, without revealing detailed visual images that cause privacy concerns. The data would be analyzed in a secure computing environment so as to enhance privacy protection. Nevertheless, privacy considerations represent a potential barrier to widespread sensor adoption, and care should be taken to not only protect patient privacy with utmost care but also convey to potential users the protective measures taken in developing sensor technologies.

When properly packaged (eg, putting the radio and depth camera in one case), such contactless sensors can be mounted on the wall and left to collect data continuously while the user simply conducts his or her daily routines as usual. There exist other contactless sensors, including the sleep monitoring functionality of the SleepScore Max and the recently released Google Nest Hub. They are only now beginning to enter the consumer market [70,71]. They offer substantial benefits, particularly among older individuals who, as they age, spend increasing amounts of time in the home; those with progressing chronic diseases; and individuals with cognitive impairment or decline.

### Wearable Sensors

Wearable sensors include anything that can collect data about a user and requires a physical proximity (usually by touch) between the user and device to operate. Wearable sensors are a widely saturated component of the current market. Often, they are sought for reasons other than their sensor functionality. Smartwatches are a particularly demonstrative example of this. Although features of the Apple Watch series 6 include pulse oximetry, actigraphy, photoplethysmography, electrocardiography, pedometry, and altimetry, its mass appeal to many stems from its ability to connect with other devices, allowing for a portable means to listen to music, access text messages, take phone calls, and check the time. Such benefits make them widely sought and already frequently used. Other continuously or regularly monitoring wearable devices include chest-strap heart rate monitors, wearable blood oxygen saturation (SpO_2_) trackers, continuous positive airway pressure (CPAP) machines, smart clothing, smart hearing aids, smart headphones, and smart glasses.

Wearable sensors are widely versatile, in part due to their portability. The tradeoff, however, is that wearable sensors need to be periodically charged and worn. For some, this may be habitual, but for others, adherence is a significant consideration that may make data collection less effective. In the context of PASC, when continuous, contextualized data collections are integral to a holistic approach to care, complementary sensor capabilities should be considered for these patients. For some users, contactless sensors may offer these complementary capabilities.

### Contactless Sensors

Contactless sensors include ambient sensors that can be strategically implemented throughout an individuals’ physical space. Such sensors can measure light, motion, vibration, pressure, and electromagnetic echoes from a distance and can be used to monitor vital signs, activity patterns, sleep quality, and activities of daily living. Newer prototypes can detect movement at such granularity as to resolve minute chest-wall motions from breaths and heartbeats [69,72,73]. With the widespread implementation of nonwearable sensors, there would be less of an onus on patients to ensure that their devices are fully charged and that their sensors are being worn consistently and correctly. Rather, contactless sensors provide a framework that makes passive monitoring possible without any cooperative efforts from the user. Such a system would allow physicians and other care providers to access continuously collected data on activity and vital signs from patients in a home or home-like environment provided patient consent to that data collection. This stands in juxtaposition to wearable sensors with which patients must actively cooperate (ie, charging, wearing) prior to data being acquired. These features of contactless sensing have made them a natural choice in monitoring ailments associated with aging, often used instead of or in conjunction with the better-established wearable sensors [74].

The added benefit of contactless sensors is the fact that some of them can be used to track multiple subjects. This cost-saving measure could be particularly well utilized in the context of PASC as acute COVID was, and continues to be, rapidly disseminated in household and nursing homes clusters, where a single sensor could be used by multiple users [75,76]. The use of contactless sensors, then, would minimize the resources necessary to effectively monitor and track PASC within high-exposure households. Nevertheless, the use of individual contactless sensors may have limited sensing range or angle, thus not offering full coverage without the installation of multiple sensors, motivating the complementary need for wearable sensors.

## Discussion

Currently, health care professionals assign a diagnosis of PASC when a patient who had previously been diagnosed with COVID-19 or who had a strong clinical suspicion of having had COVID-19 has ongoing or new symptoms of otherwise unexplainable etiology that arose after the acute COVID-19 phase. Such patients are now commonly managed in newly designated PASC clinics offering periodic professional consultation and symptom management for this heretofore poorly understood disease [77]. Before enrollment in a PASC clinic, few in-hospital tests can be conducted to further support the diagnosis of PASC. These diagnostics, including basic laboratory testing, serological screening, targeted history and physical taking, and stress testing can be viewed on the CDC website [78]. These tests are limited in their ability to quantify symptoms in waxing and waning cycles of symptom intensity. In addition to this general and unspecific approach to diagnosis, treatment for PASC, when available, relies on reactive management of symptoms. These challenges in monitoring and managing symptoms can, in part, be addressed with the widespread adoption of sensors; sensors can facilitate continuously monitoring a patient by multimodality data collection. This monitoring, in concert with analysis and interpretation of existing tools that have diagnostic power, can provide a more holistic picture of PASC and its pathophysiological progression.

Continuous sensing devices like smartwatches with even basic measurement capabilities would be instrumental in clarifying for the medical community the temporal development of PASC. Furthermore, it would be useful in differentiating symptom clusters of PASC as has recently been observed [79]. Such devices, where feasible, should be provided to patients at initial presentation with COVID-19. For patients who already have and use continuous sensing devices, a previously established baseline of vital signs would help track symptom abatement. Such deviation-from-baseline tracking has already been used to predict acute COVID-19 before the onset of clinical symptoms and has begun to be used in the context of PASC [79,80].

With regards to symptom monitoring and tracking, there is substantial utility in the use of sensors to derive context-specific symptom management. Many symptoms associated with PASC are exacerbated, and some are alleviated by performing certain tasks. Many patients complain about having difficulties performing activities of daily living (ie, climbing stairs, standing). Having quantitative time-linked, or in the case of some contactless sensors, directly activity-linked, vital sign metrics would be helpful in both clinical management and research. With context-specific data, symptom management can become pre-emptive rather than reactive. Furthermore, with quantitative measures of symptoms, occupational and physical therapists can better address difficulties in performing specific daily tasks. Additionally, while many of the signs associated with each of the above common symptoms are nonspecific, thorough cataloging of disease sign presentations will be extremely impactful in shaping management and understanding.

Ultimately, the utility of continuous sensing devices in the context of PASC has both clinical and academic/research utility. Such data collection will not only provide better-targeted care to the individual patients with persisting COVID symptoms but will also contribute to the medical understanding of how PASC manifests. Such information is critical in the data-poor arena of PASC. The use of such sensors in the monitoring of chronic diseases is not a new concept, and existing projects have been met with success. Programs have been implemented to both track and facilitate activity in individuals with chronic disease and to monitor elderly populations to facilitate aging in place [18,81-85]. These patient populations have benefitted largely from the volume of data collected to improve the quality of life for patients and to better describe malady in general. Given the global burden of the COVID-19 pandemic, a similar strategy to those already being implemented should be initiated to determine how best to allocate resources to individuals with PASC symptoms. Such investment in monitoring, especially monitoring by passive means due to the lack of extra user efforts, will take some of the burden off medical professionals and health care resources by streamlining care, allowing physicians a more holistic understanding of their patients’ medical presentations, and providing them a foundation on which to prioritize management strategies. The net result of such widespread adoption of innovative sensor technologies would be to facilitate the diminution of some of the long-term systemic drains on the medical system due to the pandemic.

There is also a social component to PASC that can be addressed with sensors. PASC has been associated with the stigma of being psychosomatic rather than physical in certain segments of the population [86]. Furthermore, many do not recognize their symptoms as being significant enough to warrant seeing a doctor or may fail to connect their symptoms with PASC [87]. With continuous sensing, the timeline associated with the onset of symptoms could be retroactively assessed by a clinician, allowing for streamlined medical care despite delays in initially seeking that care. Though the use of wearable and contactless sensors for monitoring health is still limited in its adoption, such technology offers patients and providers significant benefits in the identification and management of PASC.

Although continuous sensing promises a substantial benefit to the health care system in the context of PASC, it is worth considering the potential challenges of a widespread continuous sensing paradigm. First, as many of these technologies are still in development and their use outside of a theoretical or research framework has so far been limited, few have received FDA approval for use in clinical management. Most will likely not receive approval within any practical timeline. As such, sensor data should be used at providers’ discretion, and data should be used to augment clinical practice and not replace it. For technologies that have gone through rigorous studies, their reliability and validity could be factored into their role in clinical care and should be considered on a case-by-case basis. Many such technologies have been demonstrated to be relatively accurate when compared with a medical gold standard [69,88,89]. However, it is not advised that non-FDA-approved devices be used for the diagnosis or management of any disease without substantial clinical suspicion to corroborate. We therefore envision the role of sensors to be adjunctive to the use of existing FDA-approved technologies. We stress that any abnormal or concerning finding obtained from non-FDA-approved technologies should trigger subsequent follow-up. Second, it is important to consider how the influx of additional information will be handled by health care professionals. Sensors can quantify physiologic signs. Associating those signs with specific symptoms is important in developing a clinical picture but requires careful interpretation of the data in conjunction with clinical judgment. Sensor data do not represent a replacement for clinical intuition. Instead, sensors promise a modality by which suspicions may be corroborated and subsequent confirmatory workup can be initiated. In instances where sensor data fail to corroborate a hypothesized pathophysiologic course, it may be impossible to determine whether the sensor(s) or the algorithms that interpret the data lack sufficient sensitivity or there is a true lack of symptom(s). The inability to resolve the cause of these discrepant measurements may lead to patient anxiety. It is, therefore, important for physicians to temper these expectations in patients and to understand the limitations inherent to any given sensing system. It should be stressed to the patient that continuous sensing can be effective at offering additional evidence correlated with suspected signs and symptoms, but it is inherently limited due to the physics of sensing hardware (ie, noises, disturbances) and the abilities of algorithms (ie, fidelity achievable interpreting signals), thus should only be used to aid but never to replace clinical measurements and diagnosis. Sensors should never serve as the sole foundation of a clinical picture or clinical management without evaluating all clinical data available.

Finally, it is also important to consider barriers to widespread sensor adoption from the perspective of users. Since sensors are effective at collecting vital sign data, there is an inherent privacy concern that may prevent their use. Care must be taken to implement Health Insurance Portability and Accountability Act (HIPAA)–compliant protocols for the encryption and protection of patient information. As part of these protocols, patients should be empowered to withhold sensor data from anyone, including their health care providers, should they so choose. The responsibility of ensuring that a given sensor sufficiently prioritizes privacy will likely fall on both the manufacturer and the individual represented at the point of acquiring the sensor (ie, retail salesperson, physician). These parties must have a practical understanding of privacy measures taken to accurately convey to the potential user the risks and benefits of using the product. Furthermore, there is a challenge of equity. The cost of purchasing sensors, either by institutions or users directly, may be prohibitive. As such, providing access to these sensor technologies in regions of low socioeconomic status may be challenging. Given the demonstrated benefits of sensors, we recommend that physicians or health care systems be empowered to provide or lend sensor technologies to those patients who stand to benefit from them, particularly those patients who cannot afford sensors themselves. Short of hospital and clinic-sponsored dissemination of sensor technologies, on a federal level, allocating resources to subsidize the purchase of vital sensors for those who cannot afford them would facilitate equity in sensor utilization. In regions that lack internet connectivity or reliable access to clinics or hospitals, the lack of existing infrastructure may make sensor utilization less effective. Providing the infrastructure necessary to accommodate these sensors is itself a public health challenge and has garnered widespread attention. It is of vital importance that reliable internet and stable access to care are ensured for all. Though such approaches to addressing health care infrastructure deficits fall beyond the scope of this paper, it is of our opinion that such basic functions should be afforded to all.

Despite these challenges, with trained health care professionals making decisions with the help of continuous sensing data, management plans can be more effectively designed and implemented, and a more comprehensive academic understanding of PASC can be achieved. Such an approach to disease tracking has been used in the context of acute COVID-19, with particular emphasis on prioritizing sensors that track respiratory rate, pulse oximetry, heart rate, heart rhythm, and blood pressure [90]. With the widespread recognition of the utility of sensors in disease tracking generally, and in COVID specifically, it is imperative that there is continued and renewed investment in developing continuous sensing technologies with relatively high home deployment feasibility and more universal adoption of such technologies between clinical visits. Particularly as segments of medical practice have adopted measures to accommodate telemedicine, the importance of a passive means of garnering quantitative patient data with real-time evolution collected outside of medical establishments is difficult to overstate [91]. Furthermore, care must be made to develop rigorous analytics software to interpret raw sensor output data into actionable insights impacting clinical decisions. Laying the groundwork for such a technologically motivated health care infrastructure would provide medical professionals a plethora of data to augment existing clinical measures for targeted, individualized health care decisions with their patients.

Although PASC is, as yet, a poorly understood disease, a symptom-based approach to tracking its pathophysiology may inform management decisions. Many of the symptoms associated with PASC are relatively well-studied, and while curative treatments may not be universally applicable to target each symptom, palliative treatments that target specific symptoms may be available. With a rigorous quantification of symptoms, clinicians can strategize treatment plans, thereby mitigating wasteful hospital spending on ineffective treatments [92]. Given their variable but often substantial cost, sensor technologies, particularly novel technologies, will need to be paid for by, subsidized by, or rented out from federal, state, or private entities in order to maximize access and equitable use. However, the benefit of tracking therapeutic effects represents a huge potential for net savings. Although this paper focuses on the application of continuous unobtrusive sensors in the management of PASC, broader applications exist for the tracking of chronic diseases generally, with estimates of spending on inadequate treatment of chronic disease as high as US $2.5 billion annually for rheumatoid arthritis patients alone, representing a substantial outlet of wasted spending that these sensors can begin to address [93]. Furthermore, patients at risk of particularly debilitating disease can be identified early in their recovery and directed to appropriate resources (ie, pharmacotherapy, physical therapy, pulmonary therapy) to preempt deterioration, thereby enhancing the efficacy of intervention with early initiation [92]. In such instances where treatments are initiated, unobtrusive or contactless sensors are well-equipped to track physiological changes associated with the intervention, and thus widespread utilization of such sensors may serve as a basis for the iterative streamlining of health interventions. Interventions that yield ineffective or deleterious effects will be easily identified and removed or replaced until optimal treatment regimens can be reached.

Such an approach promises high potential for significant improvement in the quality of life for PASC patients and a more efficient utilization of health care spending. Similar symptom-tied approaches to continuous sensing will likely be of significant clinical utility in the management of other chronic diseases as well. The NIH recognizes this potential. In their recently released NIH-Wide Strategic Plan for fiscal years 2021-2025, they acknowledge the importance of being able to track patient signs across time, outside of a clinical setting [94]. The document also emphasizes the utility of “sensors that can provide continuous feedback,” particularly because of their ability to “detect underlying signs of illness and response to intervention, including medications and lifestyle changes faster than conventional methods.” Such a formal recognition of the importance of continuous sensing will be invaluable in reinforcing the academic credibility of such further study.

The COVID-19 pandemic has forced a renewed awareness of health care delivery systems and the technologies that define them. It is imperative, then, that we in the medical and engineering communities engage with the public discourse of the present moment and leverage the development, adoption, and interpretation of technologies that streamline care and yield promising health outcomes. We consider continuous sensing to be an essential component of this modern health care system. Such a goal of streamlined and optimal care necessitates interprofessional collaboration and community engagement. It is our hope that this viewpoint provides suggestions and inspiration for future interdisciplinary collaborations that facilitate the innovation of sensor technologies and the infrastructure that supports them.

## Abbreviations

CDC: Centers for Disease Control and Prevention
CPAP: continuous positive airway pressure
EEG: electroencephalogram
FDA: Food and Drug Administration
HIPAA: Health Insurance Portability and Accountability Act
NICE: National Institute for Health and Care Excellence
NIH: National Institutes of Health
PASC: postacute sequelae of SARS CoV-2
POTS: postural orthostatic tachycardia syndrome
SpO2: blood oxygen saturation
WHO: World Health Organization
